# Dietary β-Sitosterol Improves Growth Performance, Meat Quality, Antioxidant Status, and Mitochondrial Biogenesis of Breast Muscle in Broilers

**DOI:** 10.3390/ani9030071

**Published:** 2019-02-26

**Authors:** Yefei Cheng, Yueping Chen, Jun Li, Hengman Qu, Yurui Zhao, Chao Wen, Yanmin Zhou

**Affiliations:** College of Animal Science and Technology, Nanjing Agricultural University, Nanjing 210095, China; 2017205020@njau.edu.cn (Y.C.); chenyp0321@163.com (Y.C.); 2017105070@njau.edu.cn (J.L.); 2017105069@njau.edu.cn (H.Q.); 2017105068@njau.edu.cn (Y.Z.); wenage@163.com (C.W.)

**Keywords:** antioxidant capacity, broiler, β-sitosterol, growth performance, meat quality, mitochondrion

## Abstract

**Simple Summary:**

Fast growth of modern broilers induces their muscle abnormality and myopathy, and therefore could compromise meat quality attributes. Antibiotic growth promoters have been banned by European Union and restricted by other countries in livestock production due to the public concern about food safety and antibiotic resistance. The search for efficacious, environmentally friendly, safe, and consumer favorable feed additives have become a necessity to poultry meat production. β-sitosterol—the most abundant phytosterol similar to cholesterol in chemical structure—is found in several plant products. It was incorporated at four levels into broiler diets (40, 60, 80, and 100 mg/kg). Dietary β-sitosterol supplementation improved growth performance and breast muscle meat quality of broilers, and the improved meat quality may be related with the simultaneously enhanced oxidative status and mitochondrial biogenesis in the breast muscle. The β-sitosterol dietary supplementation at the level of 80 mg/kg is recommended in broilers

**Abstract:**

The present study evaluated effects of β-sitosterol on growth performance, meat quality, oxidative status, and mitochondrial biogenesis of breast muscle in broilers. One-day-old chicks were allocated to five treatments of six replicates. Broilers were fed a basal diet supplemented either with 0 (control), 40, 60, 80, or 100 mg/kg β-sitosterol for 42 days. β-sitosterol linearly and quadratically reduced feed/gain ratio, lightness_24h_ and cooking loss_24h_ in breast muscle, whereas 2, 2-diphenyl-1-picrylhydrazyl scavenging activity of breast muscle followed an opposite trend. β-sitosterol linearly decreased drip loss_24h_ and malondialdehyde content, whereas linearly increased pH_24h_, superoxide dismutase activity, and mRNA abundances of peroxisome proliferator-activated receptor γ coactivator 1α (PCG-1α) and mitochondrial transcription factor A (TFAM) in breast muscle. Compared with control, levels of β-sitosterol higher than 40 mg/kg reduced feed/gain ratio, muscular lightness_24h_, cooking loss_24h_, and malondialdehyde level, whereas increased muscular 2, 2-diphenyl-1-picrylhydrazyl scavenging activity, and mRNA abundances (except 60 mg/kg) of PCG-1α and TFAM. Eighty milligram/kilogram β-sitosterol increased muscular pH_24h_ and superoxide dismutase activity, but decreased its drip loss_24h_. Therefore, β-sitosterol could improve growth performance and meat quality, oxidative status, and mitochondrial biogenesis of breast muscle in broilers. Furthermore, supplementation level of 80 mg/kg β-sitosterol is recommended for broiler diets.

## 1. Introduction

Broiler meat is generally selected by the consumers due to its low cost and healthy nutritional profile [1]. Herein, the producers use fast growth of modern broilers to obtain more meat yield. However, the fast growth rate of broilers in turn induce their muscle abnormality and myopathy, adversely affecting meat quality attributes [2,3]. Additionally, antibiotic growth promoters have been absolutely banned by European Union and strictly restricted by other countries in livestock production due to the public concern about food safety and antibiotic resistance [4,5]. Therefore, the search for efficacious, environmentally friendly, safe, and consumer favorable feed additives have become necessary in poultry meat production systems. Dietary manipulation is one of the most common practices for the improvement of meat quality in poultry. Currently, dietary supplementation with plant-derived extracts, for example, curcumin, resveratrol, and *Artemisia annua* L., has attracted increasing attention by researchers [6,7,8]. 

Muscular oxidative status is associated with the meat quality since oxidation is one of the major issues of quality deterioration in meat [9]. Plant-derived extracts exert promising antioxidant capacity and herein could improve meat quality in poultry production [6,7,8,10]. Phytosterols are plant-derived natural steroid compounds, and mainly contain β-sitosterol, stigmasterol, campesterol, and brassicasterol. The effects of phytosterols’ application on livestock production have been recently investigated [11,12,13,14,15,16]. However, due to their mixed compositions, it is difficult to explain their mode of action and their physiological functions. β-sitosterol, the most abundant phytosterol that is similar to cholesterol in chemical structure, is naturally widespread in plant products. Extensive in vitro and clinical researches have already demonstrated that β-sitosterol exerts cholesterol-lowering [17,18,19], anticancer [20,21,22], and anti-inflammatory [18,23] effects. Furthermore, it has been reported that β-sitosterol supported the enzymatic and nonenzymatic antioxidant systems of macrophages [24], enhanced free radicals scavenging capacity of macrophages stimulated by phorbol ester [25], and attenuated oxidative damage in thymocyte exposed to irradiation [26]. In several in vivo studies, β-sitosterol was efficient in ameliorating streptozotocin- and 1,2-dimethylhydrazine-induced oxidative damage in rats [27,28]. In spite of the excellent aforementioned biological functions, information is extremely scarce regarding the effects of dietary β-sitosterol application on poultry production, and the first aim of this work was to evaluate whether dietary β-sitosterol could improve muscular oxidative status and meat quality in broilers.

Apart from oxidative status of muscle, its mitochondrial content and function is related to the meat quality as well. Mitochondrial density represents a criterion to assess muscular oxidative metabolic activity [29]. It has been reported that post-mortem muscular mitochondrion keep intact and functional [30,31], and therefore affects the process of muscle oxidative metabolism, eventually influencing meat quality [30,32]. Several studies with exciting results have demonstrated that β-sitosterol had beneficial effects on mitochondrial biogenesis and function in C2C12 myotubes and HT22 cells [33,34,35]. However, to the best of our knowledge, scarce data are available in term of whether β-sitosterol could improve muscle mitochondrial biogenesis in broilers, which was the second objective of the present study. Based on the aforementioned hypotheses, we speculated that β-sitosterol could induce beneficial consequences on broilers. Accordingly, this study aimed to investigate the effects of dietary β-sitosterol supplementation at different levels on growth performance, meat quality, and antioxidant ability, as well as mitochondrial biogenesis of breast muscle in broilers. 

## 2. Materials and Methods

### 2.1. Animals, Diets, and Experimental Design

All procedures involving animals in the present study were approved by Nanjing Agricultural University Animal Care and Use Committee, Nanjing, China (Certification No.: SYXK (Su) 2011-0036, 11 August 2015).

A total of 240 one-day-old male Arbor Acres plus broiler chicks with similar initial weights were randomly allocated into 5 treatments of 6 replicates (cages) with 8 birds per cage. Broilers in the 5 groups were fed a basal diet supplemented either with 0 (control group), 40, 60, 80, or 100 mg/kg β-sitosterol (the analyzed purity was 88.92%, and provided by Yichun Dahaigui Life Science Co., Ltd., Yichun, P. R. China) for 42 days. Chickens were reared in a temperature-controlled room, and had free access to clean water and mash feed in the 3-layer wired cages (120 cm × 60 cm × 50 cm; 0.09 m^2^ per bird). A light schedule of 23-h light and 1-h darkness in the housing room was provided. The temperature in the room was maintained 32 to 33 °C for the first 3 days, and it was gradually reduced by 3 °C per week to a final temperature of 20 °C. The basal diet was formulated according to the recommendation by NRC [36]; the composition and analysis of the grower and finisher basal diet are shown in Table 1. At 42 days, broilers were weighed on cage basis after a 12-h feed deprivation, and feed intake was recorded by cage, which was used to calculate average feed intake (ADFI), average body gain (ADG), and feed/gain ratio (F/G). Died broilers during the experimental period were also weighed for the F/G correction.

### 2.2. Sample Collection

At 42 days of age, one broiler (close to the cage average body weight) from each cage was selected. Chickens were bled from jugular vein and then euthanized by cervical dislocation and necropsied immediately. The right-side breast muscle was collected and stored immediately at 4°C for subsequent determination of meat quality including pH value, meat color, drip loss, cooking loss, and shear force. Meanwhile, a part of left-side breast muscle was sampled and frozen quickly at −80 °C for further analysis.

### 2.3. Meat Quality Determination

The breast muscle pH value was measured at 45 min (pH_45min_) and 24 h (pH_24h_) post-mortem in triplicate using a pH meter (HI9125, HANNA Instruments, Italy), as described by our recent work [38]. The color of breast muscle were determined at 45 min and 24 h post-mortem in triplicate using a handheld colorimeter (MinoltaCR-400, Konica Minolta, Tokyo, Japan), in which the L*, a*, and b* values were presented as indicators of lightness, redness, and yellowness, respectively. The drip loss of breast muscle at 24 and 48 h post-mortem was assayed in agreement with our previous trial [38]. Briefly, meat samples were trimmed to an approximately equal size, weighed, placed in a polyethylene bag, and hung at 4°C for 24 and 48 h, and the weight differences were recorded for drip loss calculation, which was expressed as g/kg of initial muscle weight. The cooking loss of pectoralis major muscle was measured at 24 h postmortem following the method of Cheng et al. (2018) [39]. In detail, meat samples were weighed and packed in polyethylene bags to be cooked by immersion in a water bath at 75 °C for 20 min. The cooked sample was cooled to room temperature and subsequently weighed again, and the weight loss was used to calculate cooking loss that was expressed as g/kg of initial muscle weight. For shear force determination, the cooked sample was cut into three strips parallel to the muscle fiber. Each strip was subsequently measured using a digital meat tenderness meter (Model C1LM3, Northeast Agricultural University, Harbin, China), and the average of the three measurements of each sample was recorded as shear force value (kilogram) of each sample. 

### 2.4. Analysis of Free Radical Scavenging Activity

The 2,2-diphenyl-1-picrylhydrazyl (DPPH) and 2,2-azino-bis (3-ethylbenzothia-zoline-6-sulphonic acid) diammonium salt (ABTS) free radicals scavenging activities were assayed following the methods described by Zhang et al. (2014) [40]. The results of DPPH and ABTS scavenging effects were expressed as percentage of radical inhibition (%). The scavenging activities of hydroxyl radical (OH^−^) and superoxide radical (O_2_^−^) were determined according to the instructions of the used commercial kits (Nanjing Jiancheng Institute of Bioengineering, Nanjing, China). The results are expressed as units (U) per milligram of protein for OH^−^ and U per gram of protein for O_2_^−^.

### 2.5. Measurement of Malondialdehyde Content and Antioxidant Enzymes Activities 

Approximately 0.4 g minced breast muscle of each sample was homogenized (w/v, 1/4) in ice-cold 154 mmol/L sterile sodium chloride solution using a homogenizer (PRO-PK-02200D, Pro Scientific, Inc., Monroe, CT, USA). Homogenate was thereafter centrifuged at 4450× *g* for 15 min at 4 °C, and the supernatant was stored at −80 °C for further analysis. Concentrations of malondiadehyde (MDA) and reduced glutathione (GSH) and activities of superoxide dismutase (SOD), glutathione peroxidase (GSH-Px), and catalase (CAT) were determined according to the manufacturer’s instructions using available commercial kits (Nanjing Jiancheng Institute of Bioengineering, Nanjing, China). Total protein concentration of breast muscle was measured by a Coomassie brilliant blue protein assay kit purchased from Nanjing Jiancheng Institute of Bioengineering (Nanjing, China). The results were normalized against total protein concentration in each sample for inter-sample comparison. 

### 2.6. mRNA Extraction and Real-time Quantitative PCR

Total RNA from breast muscle was isolated using Trizol reagent according to the instructions of manufacturer (TaKaRa Biotechnology Co. Ltd., Dalian, China). The concentration and purity of RNA was quantified with a spectrophotometer (NanoDrop 2000c, Thermo Scientific, USA). After that, RNA was immediately reverse transcribed into cDNA using the Prime Script RT Master Mix reagent kit according to the manufacturer’s protocols (TaKaRa Biotechnology Co., Ltd., Dalian, China). The primer sequences were synthesized by Invitrogen Biotechnology Co., Ltd. (Shanghai, China) and listed in Table 2. The cDNA samples were amplified with the SYBR Premix Ex TaqII Tli RNaseH Plus kit based on an ABI7500 Real-time PCR system (Applied Biosystems, Grand Island, NY, USA). Detailed procedures of real-time quantitative PCR were performed following the descriptions by our recent study [41]. Each sample was measured in triplicate, and gene expression was calculated relative to β-actin using the 2^−ΔΔCT^ method [42].

### 2.7. Mitochondrial DNA (mtDNA) Copy Number Measurement

Total DNA was extracted from frozen breast muscle samples using the universal Genomic DNA extraction kit according to the instructions provided by TaKaRa Biotechnology Co. Ltd. (Dalian, China). Its concentration was measured using a spectrophotometer (NanoDrop 2000c, Thermo Scientific, USA), and subsequently diluted to the same concentration for further real-time PCR analysis. The detailed procedure of quantitative real-time PCR for relative mtDNA content is described in the previous paragraph (2.6). The primer specific for the mtDNA (mtD-loop) was obtained from the paper of Zhang et al. (2018) [43], and presented in Table 2. β-Actin was chosen as the reference gene. The 2^−ΔΔC^^T^ method was employed to calculate the relative mtDNA copy number [41].

### 2.8. Statistical Analysis

All data were analyzed by one-way analysis of variance (ANOVA) using SPSS 19.0 for windows (SPSS Inc., Chicago, IL, USA). Polynomial contrasts were used to test the linear and quadratic effects of dietary β-sitosterol levels. Replicate was defined as an experimental unit for the trial. Tukey’s multiple range test was used to compare the means among treatments. The level of significance was *p* < 0.05 in all analyses. Results are presented as means and standard error of means. 

## 3. Results

### 3.1. Growth Performance 

Dietary β-sitosterol addition linearly (*p* = 0.034, Table 3) and quadratically (*p* = 0.006) reduced F: G in broilers during the 42-day study. Compared with the control group, β-sitosterol supplementation at levels of 60 and 80 mg/kg decreased F/G during the entire period (*p* < 0.05). However, broilers of the five treatment groups had similar (*p* > 0.05) average body gain (ADG) and average feed intake (ADFI) during the whole period. 

### 3.2. Meat Quality of Breast Muscle 

Lightness_24h_ and cooking loss in the breast muscle were linearly and quadratically decreased by increasing β-sitosterol addition (*p* < 0.05, Table 4). Moreover, β-sitosterol addition linearly increased pH_24h_ (*p* = 0.003), while linearly reduced drip loss_24h_ (*p* = 0.003) of breast muscle. Compared with the control group, broilers fed 80 mg/kg β-sitosterol exhibited a higher pH_24h_ whereas a lower drip loss_24h_ in the breast muscle (*p* < 0.05). In contrast, the inclusion of β-sitosterol higher than 40 mg/kg decreased lightness_24h_ and cooking loss of breast muscle (*p* < 0.05). However, β-sitosterol dietary supplementation did not affect redness, yellowness, or shear force of breast muscle (*p* > 0.05). 

### 3.3. Free Radical Scavenging Activity of Breast Muscle

Treatments did not affect scavenging activities of ABTS, and O_2_^−^, or OH^−^ levels in the breast muscle (*p* > 0.05, Table 5). In broilers fed the basal diet supplemented with β-sitosterol, DPPH scavenging activity was linearly (*p* < 0.001) and quadratically (*p* = 0.028) increased in breast muscle. Levels of β-sitosterol higher than 40 mg/kg increased DPPH scavenging activity of breast muscle when compared with the control group (*p* < 0.05).

### 3.4. Antioxidant Status of Breast Muscle 

The increase of dietary β-sitosterol levels linearly decreased MDA concentration (*p* < 0.001, Table 6), whereas linearly increased SOD activity (*p* = 0.023) in breast muscle. Compared with the control group, 60, 80, and 100 mg/kg inclusion of β-sitosterol reduced muscular MDA content (*p* < 0.05), and at the level of 80 mg/kg elevated muscular SOD activity was observed (*p* < 0.05). The activities of GSH-Px and CAT and GSH content in breast muscle, however, were not influenced by β-sitosterol inclusion (*p* > 0.05).

### 3.5. Mitochondrial DNA Content in the Breast Muscle

**Figure 1:**
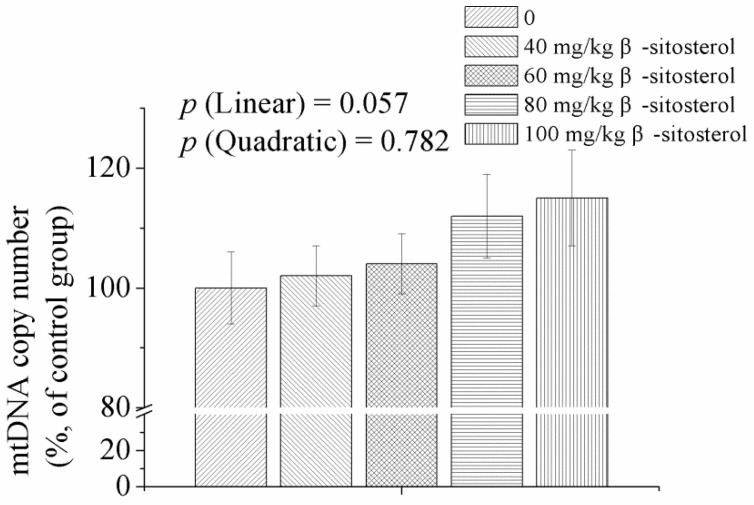
Effect of dietary β-sitosterol supplementation at different levels on genes expressions related to mitochondrial biogenesis of breast muscle in broilers. mtDNA, mitochondrial DNA. Data are shown as means and standard errors (*n* = 6).

Muscular mtDNA copy number was similar among treatments (*p* > 0.05, Figure 1). However, there was a trend of linear increase in mtDNA copy number as an effect of dietary supplementation with β-sitosterol (*p* < 0.1).

### 3.6. Gene Expressions in the Breast Muscle

**Figure 2:**
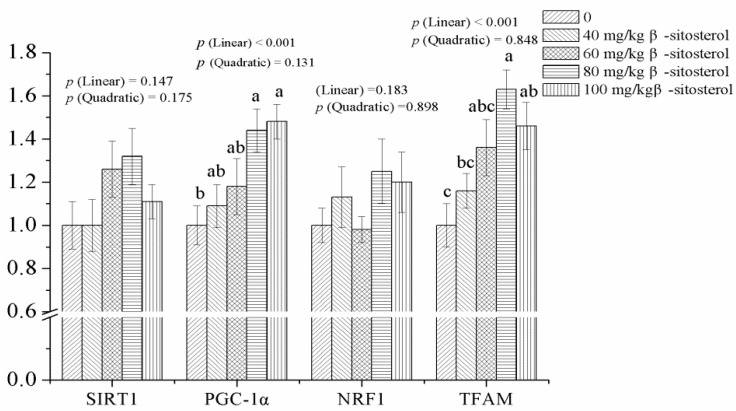
Effect of dietary β-sitosterol supplementation at different levels on genes expressions related to mitochondrial biogenesis of breast muscle in broilers. SIRT1, sirtuin 1; PGC-1α, peroxisome proliferator-activated receptor γ coactivator 1α; NRF1, nuclear respiratory factor 1; TFAM, mitochondrial transcription factor A. Data are shown as means and standard errors (*n* = 6). ^a-c^ Different letters indicate significant differences among the treatments (*p* < 0.05).

Dietary β-sitosterol supplementation linearly (Figure 2, *p* < 0.001) increased mRNA abundances of PGC-1α and TFAM in breast muscle. Broilers receiving 80 and 100 mg/kg β-sitosterol exhibited increases in PGC-1α and TFAM mRNA abundances, respectively, in the breast muscle compared with the control group (*p* < 0.05). However, muscular SIRT1 and NRF1 gene expressions were not altered by β-sitosterol addition (*p* > 0.05).

## 4. Discussion

### 4.1. Growth Performance

The information is scarce regarding the effects of β-sitosterol, the most abundant phytosterol, on growth performance in broilers. In the present study, dietary β-sitosterol supplementation at levels of 60 and 80 mg/kg reduced F/G of broilers during the 42-day study, suggesting that β-sitosterol addition could improve growth performance in broilers. Similar findings were observed by Naji et al. (2013), who reported that dietary phytosterols could improve growth performance in broilers during a 21-day experiment [14]. It has been previously demonstrated that β-sitosterol could regulate lipid metabolism, increase antioxidant capacity, and ameliorate inflammatory response [18,19,27], which could positively affect the health status of animals. These findings could, at least partially, explain the improved growth performance of broilers receiving β-sitosterol in the current study. However, controversial results on growth performance as an effect of phytosterols supplementation have also been observed in broilers [13], laying hens [15], and weaned piglets [11]. The discrepancies may be related with dosage and composition of phytosterols, but also species, environment, feeding management, etc. 

### 4.2. Meat Quality in the Breast Muscle 

Cooking loss and drip loss are parameters that are generally used to evaluate water-holding capacity of meat. It is undoubtedly that a lower pH value coupled with a weaker water-holding capacity induces liquid outflow, and loss of soluble nutrients and flavor, subsequent leading to a deteriorated meat quality [44]. In the current research, the results showed that dietary β-sitosterol supplementation at levels higher than 40 mg/kg reduced lightness at 24 h post-mortem and cooking loss, and its dosage at 80 mg/kg increased pH value whereas decreased drip loss at 24 h post-mortem in the breast muscle. Our observations are in agreement with previous studies where dietary supplementation with plant-derived extracts, such as curcumin, resveratrol, and *Artemisia annua* L., and rosemary, could improve meat quality of broilers, as illustrated by decreased cooking loss, whereas drip loss, L^*^, increased a^*^ and pH [6,7,8,45]. Furthermore, phytosterols could enhance meat quality as evidenced by improved mechanical properties of meat during a 21-day study in a recent paper [14]. The findings of our work suggested that the supplementation of dietary β-sitosterol is also an efficient way to improve meat quality of broilers. 

### 4.3. Antioxidant Capacity in the Breast Muscle

Post-mortem oxidative stability of chicken meat is related to free radical scavenging capacity of musculature and dietary antioxidants supplementation [6]. Moreno (2003) reported that β-sitosterol increased O_2_^−^ scavenging ability in phorbol ester-stimulated macrophages [25]. Consistently, our research observed that dietary β-sitosterol at levels higher than 40 mg/kg increased the DPPH radical scavenging ability in the breast muscle. This finding indicated that dietary β-sitosterol, as a potential antioxidant, improved the antioxidant ability of breast muscle in broilers, which may have attributed either to its direct capacity to neutralize stable free radicals or indirect role as a hydrogen donor [46].

Enzymatic and nonenzymatic antioxidant systems are main antioxidant defense systems and play a vital role in maintenance of redox balance. The overproduction of free radicals can be quenched by antioxidants, like GSH, and/or converted into hydrogen peroxide by SOD, and hydrogen peroxide could further be degraded to water and oxygen by GSH-Px and CAT. It has been demonstrated that β-sitosterol could enhance antioxidant enzymes activities, such as SOD, GSH-Px, and/or CAT in the cells under both normal and adverse conditions [24,25]. Broiler meat contains relative high levels of polyunsaturated fatty acids, and as a result is susceptible to free radical attack [47]. MDA is an end product of lipid peroxidation in which carbon–carbon double bonds of lipid is attacked by free radicals, and its accumulation is related with high lipid peroxidation rates. As indicated, β-sitosterol inclusion at levels higher than 40 mg/kg decreased MDA concentration of breast muscle, and its dosage at 80 mg/kg increased SOD activity of breast muscle. These findings were consistent with the results by Baskar et al. (2010), who have found that β-sitosterol could increase activities of SOD, GSH-Px, and CAT; increase GSH content; and reduce MDA concentration in the livers of rats treated with 1,2-dimethylhydrazine [21], and the findings by Naji et al. (2013), who reported that phytosterols could reduce MDA content and increase GSH concentration in the breast muscle of broilers [14]. The enhanced antioxidant enzyme activity and the reduced MDA levels were combined with increased DPPH scavenging capacity in the breast muscle, indicating that dietary β-sitosterol could improve muscle antioxidant capacity. Moreover, other plant-derived extracts, for example, curcumin, resveratrol, *Artemisia annua* L., and rosemary could increase muscular SOD and GSH-Px activities, and decrease MDA accumulation in broilers [6,7,8,45]. Muscle oxidative status is closely related with meat quality since meat oxidation including lipid peroxidation, could reduce hydrolysis sensitivity, weaken protein degradation and reduce water reservation among myofibrils, with adverse effects on several meat properties, such as cooking loss, drip loss, meat color, and pH [7]. Therefore, an improved meat quality of breast muscle as an effect of β-sitosterol addition could, at least partially, allocated to its improved antioxidant capacity. 

### 4.4. Mitochondrial Biogenesis in the Breast Muscle

PGC-1α is a key regulator in energy metabolism and controls mitochondrial biogenesis and function, which could be activated by SIR1, one of the major regulators of energy expenditure. Activated PGC-1α directly interacts with and co-activates NFR1, which targets TFAM and therefore mediates mitochondrial function. Accordingly, increased mRNA abundances of SIR1, PGC-1α, NFR1, and/or TFAM could contribute to improved mitochondrial biogenesis, and function. Mitochondrial function is related to mitochondrial content, and it can be measured quantitatively by the copy number of mtDNA. In several in vitro trials, it has been demonstrated that β-sitosterol could enhance mitochondrial biogenesis and function via increasing mitochondrial electron transport and energy demand and by activating protein kinase/PGC-1 in C2C12 myotubes, and by increasing mitochondrial membrane fluidity in HT22 cells [33,34,35]. However, in vivo studies are extremely limited regarding the effects of β-sitosterol on mitochondrial biogenesis and function. The findings of the current work illustrated that dietary β-sitosterol at levels of 80 and 100 mg/kg upregulated mRNA abundances of PGC-1α and TFAM, and numerically increased mtDNA copy number in the breast muscle, suggesting that dietary β-sitosterol could improve mitochondrial biogenesis and function in the breast muscle in broilers. These results are consistent with the available literature in which a plant-derived extract increased PGC-1α and NRF1 genes expression levels in the breast muscle of broilers [7]. It has been reported that mitochondrial content and function could influence pH decline rate of muscle post-mortem [30]. Additionally, meat color development and stability could be directly affected by mitochondrial activity [31]. Therefore, the improved mitochondrial biogenesis in the breast muscle resulting from β-sitosterol may account for the improved meat quality at some extent observed in the present study.

## 5. Conclusions

The results of our study indicated that dietary β-sitosterol was able to improve growth performance and breast muscle meat quality of broilers. β-sitosterol supplementation also enhanced antioxidant capacity and mitochondrial biogenesis of breast muscle, which in turn account for its better meat quality. Moreover, the inclusion level of 80 mg/kg β-sitosterol in broiler diet was recommended. 

## Figures and Tables

**Table 1:** Compositions and nutrient levels of the basal diet (g/kg, as fed basis unless otherwise stated).

Items	1–21 days	22–42 days
Ingredients		
Corn	570	615.2
Soybean meal	315.1	250
Corn gluten meal	34	46
Soybean oil	31	41
Limestone	12	14
Dicalcium phosphate	20	17
L-Lysine	3.4	3
DL-Methionine	1.5	0.8
Sodium chloride	3	3
Premix ^†^	10	10
Calculated nutrient levels ^‡^		
Apparent metabolizable energy (MJ/kg)	12.55	13.05
Crude protein	233	200
Calcium	10	9
Available phosphorus	4.5	3.5
Lysine	11	10
Methionine	5	3.8
Methionine + cystine	9	7.2

^†^ Premix provided per kilogram of diet: vitamin A (transretinyl acetate), 10,000 IU; vitamin D3 (cholecalciferol), 3,000 IU; vitamin E (all-rac-α-tocopherol), 30 IU; menadione, 1.3 mg; thiamin, 2.2 mg; riboflavin, 8 mg; nicotinamide, 40 mg; choline chloride, 400 mg; calcium pantothenate, 10 mg; pyridoxine·HCl, 4 mg; biotin, 0.04 mg; folic acid, 1 mg; vitamin B12 (cobalamin), 0.013 mg; Fe (from ferrous sulfate), 80 mg; Cu (from copper sulphate), 8.0 mg; Mn (from manganese sulphate), 110 mg; Zn (from zinc oxide), 60 mg; I (from calcium iodate), 1.1 mg; Se (from sodium selenite), 0.3 mg.

^‡^ Calculated according to tables of feed composition and nutritive values in China, 2012 [37].

**Table 2:** Sequences for real-time PCR primers.

Gene ^†^	Gene Bank ID	Primer Sequence, Sense/Antisense	Length (bp)
SIRT1	NM_001004767	GATCAGCAAAAGGCTGGATGGT ACGAGCCGCTTTCGCTACTAC	143
PGC-1α	AB170013.1	GACGTATCGCCTTCTTGCTC CTCGATCGGGAATATGGAGA	157
TFAM	NM_204100.1	GTGAAAGCCTGGCGAAACTG CACAGCTCAGGTTACACCGT	228
NRF1	NM_001030646.1	AAGAACACGGCGTGACTCAA TCGCTTCCGTTTCTTACCCG	274
mtD-loop	XM_015291451.1	AGGACTACGGCTTGAAAAGC	198
		CATCTTGGCATCTTCAGTGCC	
β-actin	NM_205518.1	TTGGTTTGTCAAGCAAGCGG CCCCCACATACTGGCACTTT	100

^†^ SIRT1, sirtuin 1; PGC-1α, peroxisome proliferator-activated receptor γ coactivator 1α; TFAM, mitochondrial transcription factor A; NRF1, nuclear respiratory factor 1.

**Table 3:** Effect of dietary β-sitosterol supplementation at different levels on growth performance in broilers.

Items ^†^	β-Sitosterol Level (mg/kg)					SEM *	*p*-Value	
	0	40	60	80	100		Linear	Quadratic
ADG (g/day)	52.0	52.9	53.9	53.9	53.3	0.5	0.344	0.395
ADFI (g/day)	89.4	89.9	90.0	90.2	90.5	0.9	0.710	0.959
F/G (g/g)	1.72 ^a^	1.70 ^ab^	1.67 ^b^	1.67 ^b^	1.70 ^ab^	0.01	0.034	0.006

^†^ ADG, average daily gain; ADFI, average daily feed intake; F/G, feed/gain ratio. * SEM, total standard error of means (*n* = 6). ^a, b^ Means within a row with different superscripts are different at *p* < 0.05.

**Table 4:** Effect of dietary β-sitosterol supplementation at different levels on meat quality of breast muscle in broilers.

Items	β-Sitosterol Level (mg/kg)					SEM *	*p*-Value	
	0	40	60	80	100		Linear	Quadratic
Lightness_45min_	40.9	40.2	39.9	40.1	39.7	0.2	0.113	0.556
Redness_45min_	7.29	7.22	7.31	7.68	7.49	0.15	0.440	0.964
Yellowness_45min_	18.6	18.2	18.5	18.3	18.1	1.39	0.669	0.970
Lightness_24h_	44.9 ^a^	43.3 ^ab^	40.9 ^b^	41.4 ^b^	41.8 ^b^	0.4	0.001	0.014
Redness_24h_	7.46	7.60	7.85	7.72	7.91	0.19	0.471	0.870
Yellowness_24h_	18.4	19.2	19.4	19.0	18.8	0.3	0.721	0.263
pH_45min_	6.46	6.52	6.49	6.54	6.53	0.02	0.284	0.630
pH_24h_	5.74 ^b^	5.79 ^ab^	5.81 ^ab^	5.89 ^a^	5.88 ^ab^	0.02	0.003	0.614
Drip loss_24h_ (g/kg)	32.8 ^a^	31.5 ^ab^	30.4 ^ab^	27.9 ^b^	29.9 ^ab^	0.5	0.003	0.173
Drip loss_48h_ (g/kg)	47.6	48.8	47.7	43.4	46.4	0.9	0.201	0.956
Cooking loss (g/kg)	196 ^a^	176 ^ab^	151 ^b^	153 ^b^	164 ^b^	4	0.001	0.003
Shear force (kg)	3.10	2.62	2.94	2.32	2.60	0.17	0.317	0.667

* SEM, total standard error of means (*n* = 6), ^a, b^ Means within a row with different superscripts are different at *p* < 0.05.

**Table 5:** Effect of dietary β-sitosterol supplementation at different levels on free radical scavenging activity of breast muscle in broilers.

Items ^†^	β-Sitosterol Level (mg/kg)					SEM *	*p*-Value	
	0	40	60	80	100		Linear	Quadratic
DPPH (%)	13.2 ^b^	14.6 ^ab^	16.3 ^a^	17.1 ^a^	16.3 ^a^	0.4	<0.001	0.028
ABTS (%)	56.2	54.7	56.1	57.8	56.9	0.4	0.141	0.645
O_2_^−^ (U/mg protein)	0.92	0.94	0.90	0.99	1.00	0.02	0.225	0.764
OH^−^ (U/g protein)	16.3	17.3	16.1	17.7	17.8	0.4	0.163	0.558

^†^ DPPH, 2,2-diphenyl-1-picrylhydrazyl; ABTS, 2,2-azino-bis-(3-ethylbenzothiazoline-6-sulphonic acid), diammonium salt; O_2_^−^, superoxide radical. OH^−^, hydroxyl radical. * SEM, total standard error of means (*n* = 6). ^a, b^ Means within a row with different superscripts are different at *p* < 0.05.

**Table 6:** Effect of dietary β-sitosterol supplementation at different levels on antioxidant status of breast muscle in broilers.

Items ^†^	β-Sitosterol Level (mg/kg)					SEM *	*p*-Value	
	0	40	60	80	100		Linear	Quadratic
MDA (nmol/mg protein)	0.61 ^a^	0.51 ^ab^	0.35 ^bc^	0.22 ^c^	0.23 ^c^	0.04	<0.001	0.200
GSH (mg/g protein)	2.06	1.94	1.85	2.61	2.63	0.15	0.106	0.382
GSH-Px (U/mg protein)	2.62	2.85	2.97	3.44	3.39	0.23	0.230	0.917
SOD (U/mg protein)	47.7 ^b^	50.3 ^ab^	54.0 ^ab^	59.7 ^a^	54.0 ^ab^	1.4	0.023	0.185
CAT (U/mg protein)	0.21	0.20	0.20	0.26	0.25	0.01	0.165	0.583

^†^ MDA, malondialdehyde; GSH, reduced glutathione; GSH-Px, glutathione peroxidase; SOD, superoxide dismutase; CAT, catalase. * SEM, total standard error of means (*n* = 6). ^a–c^ Means within a row with different superscripts are different at *p* < 0.05.
